# High prevalence of *Staphylococcus aureus* and methicillin-resistant *S. aureus*colonization among healthy children attending public daycare centers in informal settlements in a large urban center in Brazil

**DOI:** 10.1186/1471-2334-14-538

**Published:** 2014-10-06

**Authors:** Eneida Dias Vianna Braga, Fábio Aguiar-Alves, Maria de Fátima Nogueira de Freitas, Monique Oliveira de e Silva, Thami Valadares Correa, Robert E Snyder, Verônica Afonso de Araújo, Mariel Asbury Marlow, Lee W Riley, Sérgio Setúbal, Licínio Esmeraldo Silva, Claudete Aparecida Araújo Cardoso

**Affiliations:** Departamento Materno Infantil, Avenida Marques de Paraná, 303, 3° andar, School of Medicine, Programa de Pós-graduação em Ciências Médicas, Fluminense Federal University, Niterói, Rio de Janeiro Brazil; Laboratório Universitário Rodolpho Albino, Programa de Pós-graduação em Patologia, Fluminense Federal University, Rua: Mário Viana, 523, Santa Rosa - Niterói– RJ CEP, Niterói, Rio de Janeiro 24241-002 Brazil; Division of Epidemiology, 530E Li Ka Shing Center, University of California, School of Public Health, Berkeley, CA 94720 USA

**Keywords:** Community-associated, Methicillin-resistant, *Staphylococcus aureus*, Nasal colonization, Risk factors, Slum, Informal settlements, *Favelas*

## Abstract

**Background:**

In the past decade methicillin-resistant *Staphylococcus aureus* (MRSA) has become increasingly prevalent in community settings. Attending a daycare center (DCC) is a known risk factor for colonization with MRSA. Brazil operates free, public DCCs for low-income families, some of which are located in census tracts defined by the Brazilian Census Bureau as informal settlements (*aglomerados subnormais*, AGSN). Physical and demographic characteristics of AGSNs suggest that *S. aureus* colonization prevalence would be higher, but little is known about the prevalence of MRSA in these settings.

**Methods:**

We conducted a cross-sectional study to assess risk factors for *S. aureus* and MRSA colonization among children attending DCCs located in AGSN vs non-AGSN. Nasal swabs were collected from children aged three months to six years in 23 public DCCs in Niterói, Brazil between August 2011 and October 2012.

**Results:**

Of 500 children enrolled in the study, 240 (48%) were colonized with *S. aureus* and 31 (6.2%) were colonized with MRSA. Children attending DCCs in AGSNs were 2.32 times more likely to be colonized with *S. aureus* (95% CI: 1.32, 4.08), and 3.27 times more likely to be colonized with MRSA than children attending non-AGSN DCCs (95% CI: 1.52, 7.01), adjusted for confounding variables.

**Conclusion:**

*S. aureus* and MRSA colonization prevalence among children attending DCCs in informal settlement census tracts was higher than previously reported in healthy pre-school children in Latin America. Our data suggest that transmission may occur more frequently in DCCs rather than at home, highlighting the importance of DCCs in AGSNs as potential MRSA reservoirs. This finding underscores the importance of local epidemiologic surveillance in vulnerable AGSN communities.

**Electronic supplementary material:**

The online version of this article (doi:10.1186/1471-2334-14-538) contains supplementary material, which is available to authorized users.

## Background

Present in the nares, but also in the throat, axilla, groin, perineum, and vagina, *Staphylococcus aureus* is the most commonly isolated bacterial pathogen in humans. It is responsible for a number of infections, ranging from uncomplicated skin and soft-tissue infections such as boils, carbuncles, and abscesses, to more severe invasive illnesses, including empyema, septic arthritis, pyomyositis, osteomyelitis, necrotizing fasciitis, pneumonia, endocarditis, and septicemia [1]. *S. aureus* nasal colonization prevalence in the general population worldwide has been estimated to be between 20-40% [2]. Previous colonization is the most important risk factor for subsequent infection [3]. Methicillin-resistant *Staphylococcus aureus* (MRSA) is resistant to all beta-lactam antibiotics, complicating the clinical management of MRSA infections. Traditionally MRSA infections have occurred in healthcare (HC) settings, but the emergence of community-associated MRSA (CA-MRSA) infection in the past decade has become a global public health threat [4]. More recently, increasing rates of cross-transmission between hospitals and communities have hampered the distinction between CA and HC acquired infections [5].

CA-MRSA was first described in specific sub-populations: Aboriginal Australians, indigenous North Americans, athletic teams, military recruits, prison inmates, and children attending daycare centers (DCC) [5]. Attempts to definitively identify risk factors for colonization or community reservoirs of MRSA have had varying levels of success. Shared characteristics of these sub-populations that could facilitate transmission include crowding, frequent skin-to-skin contact, participation in activities that result in compromised skin surfaces, sharing of personal items, barriers to adequate hygiene, and frequent antibiotic exposure [6].

Many of these risk factors are also characteristics that manifest in informal human settlements or slums, colloquially referred to as *favelas* in Brazil. *Favelas* are characterized by concentrated poverty, inadequate shelter, poor physical environmental conditions, political and social exclusion, and generation-spanning poverty and violence, all of which contribute to a wide variety of adverse health outcomes [7]. Despite this knowledge, no studies have been conducted to explicitly compare *S. aureus* colonization prevalence and risk factors for colonization between those living within and outside of slums.

Epidemiologic data describing the prevalence and transmission of CA-MRSA in Brazil are scarce. However, some studies and reports have indicated that the prevalence of CA-MRSA is increasing [8, 9]. The global incidence of HC-MRSA infection in adults has decreased since 2005, but a recent study by Iwamoto *et al.* indicated an increase in the rate of CA-MRSA among children in United States involving selected counties in nine states [10].

Healthy children attending DCCs are more likely to become infected with MRSA than children who do not attend DCCs [11]. In Brazil, the government subsidizes and operates free-public DCCs. Children attending these DCCs frequently come from families without the financial means to send their children to private DCCs. This study was conducted in Niterói, Brazil, located in the greater metropolitan area of Rio de Janeiro. According to the 2010 census, 3,084 (46.4%) of 6,642 children attending DCCs in Niterói attended public DCCs. In 2013, Niterói had the seventh highest Human Development Index (HDI) of the 5,565 Brazilian cities surveyed [12]. This index combines life expectancy, education, and income to rank levels of human development across different geographic areas. Despite these favorable indicators, inequalities persist in the municipality as 16% of the population resides in informal settlements, operationally defined by the Brazilian census bureau as subnormal agglomerations (AGSN).

The aims of this study were to assess and compare clinical, epidemiologic and geographic risk factors for colonization with *S. aureus* and MRSA, and compare the prevalence of nasal colonization among children attending public DCCs located in informal and non-informal settlements in Niterói.

## Methods

### Study design and study population

**Figure 1:**
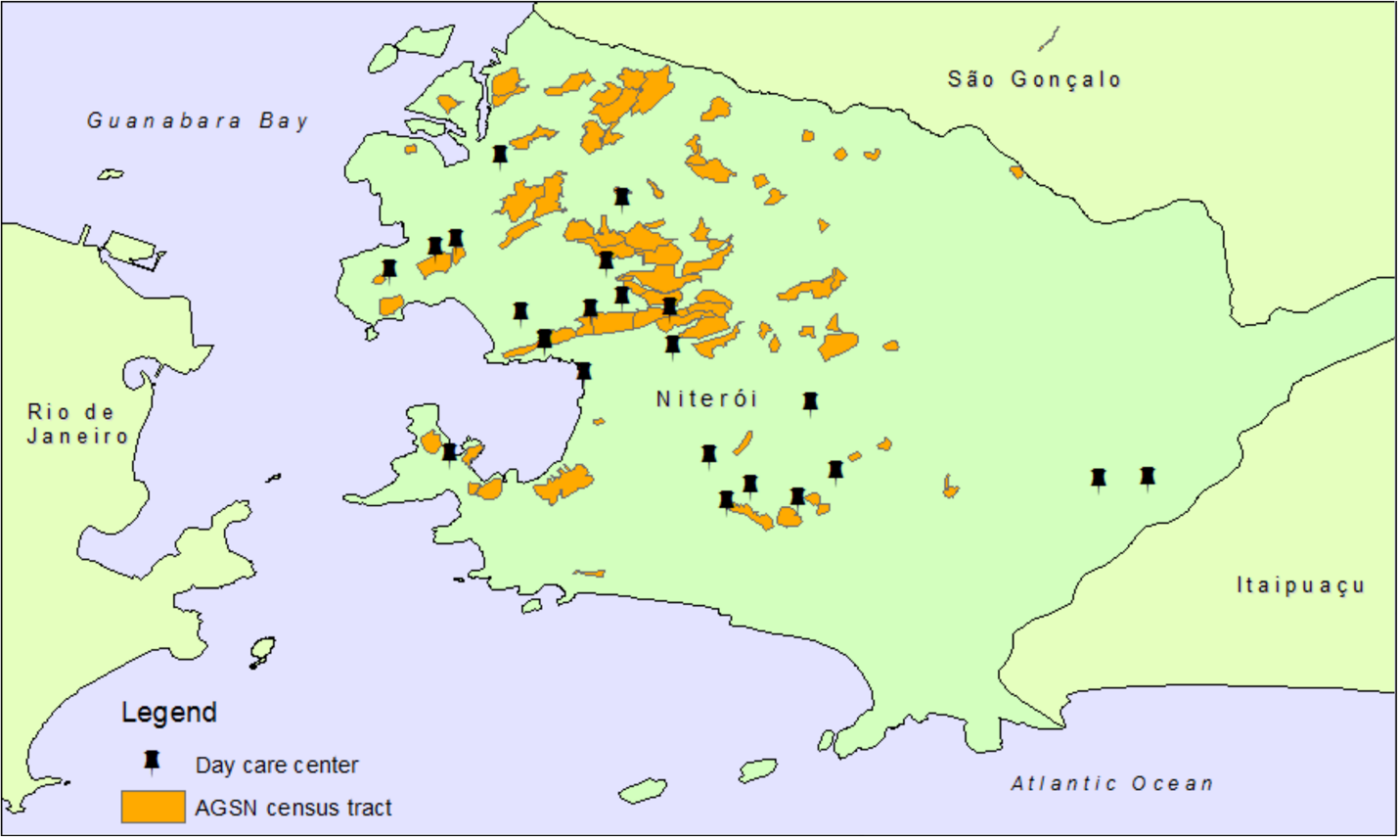
**Map of Niterói indicating location of the twenty-three daycare centers (DCC) included in the study.** Informal settlement (aglomerado subnormal, AGSN) census tracts as identified in the 2010 Brazilian Census are denoted in orange.

We performed a cross-sectional study in which researchers consecutively recruited study participants as they arrived or left the DCC until at least 15% of the selected DCC’s population had been sampled. Five hundred children (16.2% of the public DCC population), three months to six years of age, attending 23 of 30 public DCCs in Niterói, Brazil were enrolled between August 2011 and October 2012 (Figure 1). Seven public DCCs were excluded because they were too dangerous (6) or had serious barriers to obtaining informed consent (1). Optimal sample size was calculated according to the total number of children attending public DCCs in Niterói in 2010 (3,084), setting power to 0.8, with an a priori estimate of 5% MRSA prevalence.

### Patient recruitment and data collection

Researchers visited DCCs at the time of a child’s arrival (8 AM) or departure (5 PM). After a brief discussion of the study’s risks, benefits, and confidentiality, investigators obtained informed consent from legal guardians. Medical histories in addition to demographic and socioeconomic characteristics were obtained from parents or guardians at the time of sample collection through use of a standardized questionnaire. Children’s enrollment histories were excerpted from DCC records. All collections at an individual DCC were completed on the same day, excepting one during which two different collections were conducted in June and August 2012.

### Phenotyping and genotyping Staphylococcus aureus isolates

Cultures were obtained by one full rotation of a sterile swab in each of the anterior nares and transported in Stuart Transport Medium (Copan, Brescia, Italy) to the laboratory within six hours of collection for immediate processing. Swabs were streaked onto blood agar plates and incubated at 37°C for 24 h to isolate single colonies. We tested all mannitol-fermenting colonies that exhibited yellow pigmentation for susceptibility to oxacillin and cefoxitin by disk-diffusion in accordance with standards described by the Clinical Laboratory Standards Institute [13]. The presence of the *mec*A gene was determined by PCR analysis in all oxacillin or cefoxitin resistant isolates as described by Swenson *et al.*[14].

### Geographic localization of DCCs and patient residences

Geographic location of a DCC was determined by the presence of the DCC in census tracts identified by the Brazilian Census Bureau (Instituto Brasileiro de Geografia e Estatística (IBGE)) in the 2010 Brazilian Census. The IBGE defines informal settlements with the unique term “subnormal agglomerations” (*aglomerados subnormais,* AGSN). These are settlements made up of a minimum of 51 housing units that also fulfill the following criteria: 1) illegal occupation of the land or 2) receipt of land title in the previous ten years and either being constructed independent of municipal regulatory agencies or experiencing a general scarcity of public services.

Participant residences were geocoded by address and mapped with IBGE census tracts in ArcGIS 10 (Esri, Redlands, CA). Geocoded addresses were intersected with the official 2010 Census tract layer to determine census tract of residence (ftp://geoftp.ibge.gov.br/malhas_digitais/censo_2010/aglomerados_subnormais/shape/). Participants whose residences were mapped inside or within 50 m of the limits of an AGSN census tract were considered as living in an AGSN. The 50 m limit was incorporated to adjust for two characteristics of AGSN addresses: many residents use a collective address just outside the AGSN limits to receive correspondence and residences are often mapped to the last available street number just outside the AGSN border since many street numbers within AGSNs remain unofficial.

### Statistical analysis

Analyses were conducted on individuals colonized with any type of *S. aureus* (inclusive of MRSA) and separately among those colonized with MRSA. Data analyses were conducted with Stata v.12.1 (StataCorp, College Station, TX). Continuous demographic and socioeconomic characteristics of AGSN and non-AGSN DCC attendees were compared with a Student’s t-test, while categorical variables of these groups were compared with the Pearson’s chi-squared test (Table 1). Risk factors for *S. aureus* and MRSA colonization were compared with Pearson’s chi-squared test (Table 2), and a multivariable logistic regression model was constructed in a reverse step-wise fashion to analyze risk factors for *S. aureus* carriage, beginning with all demographic and population characteristics from Table 2, and maintaining variables with *p* < 0.20 in the final multivariable model. Variables included in the final logistic model were presented as adjusted odds ratios (AOR), and results for risk factors were presented as odds ratios (OR) and 95% CIs (Table 3). A small number of cases (31) precluded multiple logistic regression analysis to assess MRSA risk factors. Unadjusted OR were presented in Table 4.

**Table 1:** **Demographic characteristics comparing children attending public daycare centers in Niterói, Brazil in aglomerados subnormais (AGSN) and non-AGSN census tracts**

		Total population (n = 500)*	Non-AGSN (n = 412)		AGSN (n = 88)		
			n		n		*p*-value
*S. aureus* colonization		240 (48%)		182 (44%)		58 (66%)	< 0.001
Methicillin-susceptible *S. aureus* colonization		209 (42%)		163 (40%)		46 (52%)	0.028
Methicillin-resistant *S. aureus* colonization		31 (6.2%)		19 (5%)		12 (14%)	0.001
Gender			412		88		0.990
	Male	256 (51%)		211 (51%)		45 (51%)	
	Female	244 (49%)		201 (49%)		43 (49%)	
Ethnicity			382		81		0.132
	White	141 (28%)		122 (30%)		19 (22%)	
	Non-white	322 (64%)		260 (63%)		62 (70%)	
Mean age in years (standard deviation)		4.02	87	3.83	407	4.07	0.138
		(1.35)		(1.38)		(1.34)	
Mean time enrolled at DCC in months (standard deviation)		17.12	85	18.48	389	16.83	0.302
		(13.33)		(14.80)		(12.98)	
ß-Lactam antibiotic use in the previous thirty days			387		83		0.596
	Yes	78 (16%)		58 (14%)		20 (23%)	
	No	392 (78%)		329 (80%)		63 (72%)	
Hospitalization in the previous twelve months			386		88		0.454
	Yes	48 (10%)		41 (10%)		7 (8%)	
	No	426 (85%)		345 (84%)		81 (92%)	
Cohabitant hospitalized in the previous twelve months			411		88		0.800
	Yes	49 (10%)		41 (10%)		8 (9%)	
	No	450 (90%)		370 (90%)		80 (91%)	
Cohabitant is an employee at a health care facility			400		88		0.280
	Yes	42 (8%)		37 (9%)		5 (6%)	
	No	446 (89%)		363 (88%)		83 (94%)	
Mother has not completed primary education (1–8 years)			362		83		0.091
	No	267 (53%)		224 (54%)		43 (49%)	
	Yes	178 (36%)		138 (33%)		40 (45%)	
Household income less than two times minimum wage			362		80		0.335
	No	119 (24%)		268 (65%)		55 (63%)	
	Yes	323 (65%)		94 (23%)		25 (28%)	
More than five household co-habitants			412		88		0.643
	No	340 (68%)		282 (68%)		58 (66%)	
	Yes	149 (30%)		130 (32%)		30 (34%)	
Shared fomites at home (bed, clothing, towels)			411		88		0.802
	Yes	152 (30%)		127 (31%)		26 (30%)	
	No	346 (69%)		284 (69%)		62 (70%)	
Time sampled (months after initial sampling)			412		88		< 0.001
	≤6 m	131 (26%)		131 (32%)		47 (53%)	
	>6 m	281 (56%)		281 (68%)		41 (47%)	
Residence located within 50 m of AGSN census tract			294		62		0.001
	No	205 (41%		181 (44%)		24 (27%)	
	Yes	151 (30%)		113 (27%)		38 (43%)	

*May not total 100% as data reporting for some characteristics were incomplete.

**Table 2:** **Comparison of demographic characteristics associated with** ***S. aureus*** **and methicillin-resistant** ***S. aureus*** **(MRSA) nasal colonization among children attending public daycare centers in Niterói, Brazil**

Risk factor		***S. aureus***nasal carriage			MRSA carriage		
		Yes	No	*p*-value	Yes	No	*p*-value
Gender	Male	121	135	0.736	13	243	0.287
	Female	119	125		18	226	
Older than 36 months	No	55	86	0.011	8	133	0.760
	Yes	185	174		23	336	
Attending DCC for more than 12 months	No	93	135	0.002	11	217	0.195
	Yes	136	110		19	227	
ß-Lactam antibiotic use in the previous thirty days	No	180	212	0.057	10	375	0.003
	Yes	45	33		17	68	
Hospitalization in the previous twelve months	No	196	57	0.599	28	398	0.932
	Yes	24	230		3	45	
Cohabitant hospitalized in the previous twelve months	No	219	231	0.440	26	424	0.223
	Yes	21	28		5	44	
Cohabitant is an employee at a health care facility	No	211	235	0.969	31	415	0.077
	Yes	20	22		0	415	
Mother has not completed primary education (1–8 years)	No	76	102	0.088	11	167	0.936
	Yes	136	131		17	250	
Household income less than two times minimum wage	No	151	172	0.597	20	303	0.839
	Yes	59	60		8	111	
More than five household cohabitants	No	167	173	0.060	18	322	0.221
	Yes	67	93		13	147	
Shared fomites at home (bed, clothing, towels)	No	67	86	0.222	9	144	0.839
	Yes	172	174		22	324	
Daycare center located in aglomerado subnormal (AGSN) census tract	No	182	230	< 0.001	19	393	< 0.001
	Yes	58	30		12	76	
Time sampled (months after initial sampling)	≤ 6 m	100	78	0.006	15	163	0.125
	> 6 m	140	182		16	306	
Residence located within 50 m of AGSN census tract	No	102	103	0.792	14	191	0.553
	Yes	73	78		8	143

**Table 3:** **Risk factors for** ***Staphylococcus aureus*** **colonization among children attending public daycare centers (DCC) in Niterói, Brazil. AORs are presented for variables included in the final model**

Risk factor for S. aureus	OR	95% CI	AOR	95% CI
Older than 36 months	1.26	1.11 - 1.45	1.32	1.12 - 1.56
Attending DCC for more than 12 months	1.02	1.01 - 1.04	-	-
Mother has unfinished primary education (1–8 years)	1.39	0.95 - 2.04	1.55	1.00 - 2.42
DCC located in AGSN	2.44	1.51 - 3.96	2.32	1.32 - 4.08
Time sampled (months after initial sampling)	0.94	0.90 - 0.97	0.94	0.90 - 0.99
Residence located within 50 m of AGSN	0.95	0.62 - 1.44	-	-

**Table 4:** **Risk factors for methicillin-resistant** ***Staphylococcus aureus*** **colonization among children attending public daycare centers (DCC) in Niterói, Brazil**

Risk factor	OR	95% CI
ß-Lactam antibiotic use in previous thirty days	3.24	1.42 – 7.39
Cohabitant is employee at health care facility*	-	-
More than five household members	1.22	0.96 - 1.56
DCC located in AGSN	3.27	1.52 - 7.01
Time sampled (months after initial sampling)	0.92	0.86 - 0.99
Residence located within 50 m of AGSN	0.76	0.31 - 1.87

*No one colonized with MRSA had a family member who was an employee at a health care facility.

The Ethics Committee at Fluminense Federal University approved this study; parents or legal guardians of all participants provided written informed consent.

## Results

**Figure 2:**
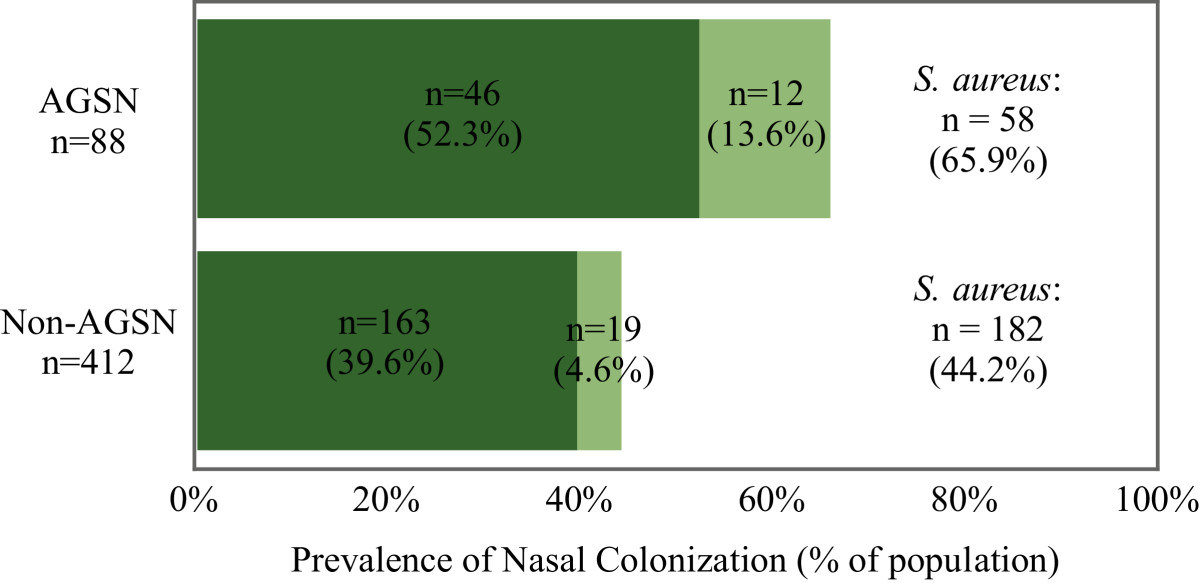
**Methicillin-resistant** ***Staphylococcus aureus*** **(MRSA) and methicillin-susceptible** ***Staphylococcus aureus*** **(MSSA) colonization prevalence in public daycare centers located in informal settlements (AGSN) and non-AGSN census tracts in the municipality of Niterói, Brazil.**

Of 23 public DCC we surveyed, five were located in AGSN and 18 were located in non-AGSN census tracts. Of the seven DCCs not sampled, six were located in AGSN census tracts. Table 1 shows a comparison of demographic and clinical characteristics of AGSN and non-AGSN DCC populations. Eighty-eight of the 500 children (17.6%) attended DCCs that were located in AGSN census tracts. Nasal carriage of all *S. aureus* types was detected in 240 subjects (48%), and MRSA nasal carriage (a subset of *S. aureus*) was detected in 31 (6.2%) of 500 subjects (Figure 2). In AGSN DCCs, 58 (65.9%) of 88 children were colonized with *S. aureus*, while 12 (13.6%) were colonized with MRSA. In contrast, in non-AGSN DCCs, 182 (44.2%) of 412 were colonized with *S. aureus* (*p* < 0.001), and 19 (4.6%) were colonized with MRSA (*p* = 0.001). In three DCCs we found *S. aureus* colonization prevalence above 80%. Two of these were located in AGSN census tracts. Prevalence of nasal colonization for DCCs located in non-AGSN census tracts ranged from 0–10.5% for MRSA and 9% to 80.7% for *S. aureus*, while in AGSN DCCs, nasal colonization ranged from 0 to 21.4% for MRSA and 41.1 – 92.8% for *S. aureus*.

The mean age at admission to the study was four years (standard deviation: 1.3). The overall study population was 51.2% male. Seventy-eight (15.6%) subjects reported use of antibiotics in the 30 days prior to their interview.

A bivariate analysis of risk factors for colonization with *S. aureus* and MRSA is presented in Table 2. Of 240 children colonized with *S. aureus*, 24 (10%) had been hospitalized or undergone a surgical procedure in the previous 12 months, while 24 (9.2%) of those not colonized were hospitalized (*p* = 0.599) (Table 2). An association was seen between beta-lactam antibiotic use and colonization with both *S. aureus* (*p* = 0.057) and MRSA (*p* = 0.003)*.* Of 31 children colonized with MRSA, three (9.7%) reported a previous hospitalization or invasive surgical procedure in the immediately preceding 12 months, while 45 (9.6%) of those without MRSA carriage reported this history (*p* = 0.932). No association was observed between those living in or within 50 m of an AGSN and *S. aureus* (*p* = 0.792) or MRSA (*p* = 0.553) colonization. Amount of time a child had been enrolled in a DCC was not correlated with age; these variables only shared 33% of their variability (*p* = 0.577). Those using beta-lactam antibiotics were 3.24 times more likely to be colonized with MRSA (95% CI: 1.42-7.39).

Logistic regression indicated that when controlling for age, mother’s education, and the small increase in odds of colonization in those sampled later during the study period, children attending public DCCs in AGSN census tracts were 2.32 times more likely to be colonized with *S. aureus* (95% CI: 1.32- 4.08) than children attending DCCs outside of AGSNs (Table 3). Bivariate analysis indicates that children attending AGSNs in DCCs were 3.27 times more likely to be colonized with MRSA than children attending non-AGSN DCCs (95% CI: 1.52-7.01) (Table 4).

## Discussion

The overall *S. aureus* nasal colonization prevalence was 48% in Niterói’s public DCC population. This is higher than those described in other studies of healthy South American children attending DCCs: 31.1% (371/1192) were colonized in a 2005 Brazilian study [15]. 31% (98/316) in Argentina (2008), [16] and 38.5% (40/104) in Colombia (2009) [17].

Comparing our DCC MRSA colonization prevalence with other studies conducted in DCCs, a higher prevalence, was found in Asia: 9.3% (40/428) in South Korea in 2008, [18] and 7.8% (473/6057) in Taiwan between 2005–2008 [19]. A recent meta-analysis of 23 cross-sectional studies in healthy children from studies conducted on four different continents (aggregated across DCCs, schools, and outpatient clinical checkups) between 2000 and 2010 produced a summary prevalence of MRSA colonization of 2.3% (95% CI: 1.8-2.7) [20]. We found a prevalence (6.2%) that was five times higher than in the aforementioned Brazilian study, conducted in the DCC population of Goiania, which found a prevalence of 1.2% [15]. This large difference in MRSA colonization could be because Niterói has a much larger AGSN population (24,286) than Goiânia (1,066). Niteroi is also immediately proximal to Rio de Janeiro, an international tourist, shipping, and economic hub.

Two hundred sixteen (90%) of 240 *S. aureus* isolates and 28 (90.3%) of 31 MRSA isolates were classified as community-associated because the children had not undergone surgery or hospitalization in the previous twelve months. When coupled with the absence of an association between colonization and residence within an AGSN, the high prevalence of MRSA and *S. aureus* in DCCs suggests that DCCs may serve as reservoirs for community-associated colonization. Such an observation underscores the need for epidemiologic surveillance in informal settlements, which our findings indicate contribute substantially to an increased risk of MRSA colonization.

In 2005, Chatterjee *et al*. reported a *S. aureus* colonization prevalence of 51% in children living in informal settlements in India but the authors did not explicitly discuss informal settlements as a risk factor for colonization [21]. This is lower than our estimate in AGSNs in Niteroi (65.9%), but higher than our aggregate estimate of colonization (48%). Our findings highlight the importance of an intimate understanding of the local context when reporting disease rates. A failure to disaggregate data by type of neighborhood would have obscured the higher risk of colonization among those living in such settlements.

Income inequality is highly correlated with *S. aureus* methicillin resistance, [22] and Brazil has one of the highest Gini Index values in the world (0.6 in 2010) [23]. This phenomenon manifests itself in the AGSN DCCs in our study, with a higher MRSA prevalence (13.6%) than non-AGSN DCCs (4.6%). Furthermore, children who attend public DCCs located in AGSN census tracts are exposed to greater social segregation than children at public DCCs outside of AGSNs. Children attending AGSN DCCs usually come from the AGSN in which that DCC is located, whereas non-AGSN DCC attendees come from all over the city. Children at AGSN DCCs spend all of their time in the same AGSN: daycare in addition to their home life, interacting with the same people. However, we did not observe an association between living in an AGSN and colonization, despite the association between attending an AGSN DCC and living in an AGSN.

Other isolated populations such as aboriginal communities in Australia, Maori communities in New Zealand, Native American and Alaskan Inuit populations in the United States and Canada, Bedouin children in Israel, and incarcerated populations experience relatively higher MRSA colonization prevalence than the general population [11, 24–28]. These populations are more likely to experience conditions associated with poverty, including but not limited to overcrowding and poor access to health care. AGSNs are similarly socially isolated communities. Residents are often stigmatized, and access into and out of some communities is extremely limited due to poor infrastructure and high rates of intrapersonal violence compared to surrounding areas. Another possibility is that a synergistic relationship exists between *S. aureus* and human populations in semi-isolated communities; more frequent contact between the same people increases the frequency of opportunities for horizontal gene transfer, which could lead to drug resistance and proliferation of the bacteria within closed communities.

There was no difference in the average number of children per DCC in AGSN (108 children per DCC) and non-AGSN (107) DCCs. These data suggest that overcrowding may not contribute to the spread of *S. aureus* or MRSA in this setting. However, we were unable to obtain floor plans of DCCs and calculate the number of children per unit area so this finding should be interpreted with caution. The same governing body administers both AGSN and non-AGSN DCCs, so the principal difference between these two types of DCC is likely to be their physical location.

We tested for temporal colonization patterns and found a slight negative trend in colonization. For each month of enrollment, there was a 6% reduction in risk of colonization. We were unable to test for seasonal trends because the summer months (January – March) correspond to DCC holidays.

Beta-lactam antibiotic use was a relatively strong risk factor (OR: 3.24, 95% CI: 1.42-7.39) for MRSA colonization. This relationship was not maintained when stratifying by AGSN (OR: 1.21, 95% CI: 0.29, 5.09) and non-AGSN (OR: 4.88, 95% CI (1.74, 13.68) status, but this may be due to the small number of those colonized with MRSA; only 19 were colonized in non-AGSNs compared to 12 in AGSNs.

While geocoding of addresses for health data is a commonly employed and useful tool, difficulties remain in specifically mapping the residences of those living in informal settlements [29]. Existing maps of Niterói’s AGSNs are of extremely poor quality. Despite the fact that our attempts to geocode resulted in successful mapping of approximately 70% of addresses, we should be cautious in our interpretation of these geocoded data. Many AGSN residents’ addresses mapped immediately outside of the AGSN because that is the address they report to receive mail. Furthermore, existing roads near AGSNs have numerous addresses aggregated at the AGSN border. Future attempts to geocode addresses in informal settlements could include more precise techniques to confirm addresses, or the incorporation of novel mapping techniques to verify a resident’s domicile.

Additional limitations of this study include the potential bias that could result from the exclusion of children whose parents or guardians were not present to provide consent. In addition, some guardians declined to participate due to time constraints. However, these participants represented a small minority of those invited to participate. Enrollment statistics collected by investigators from 2012 indicate that we were able to sample 88/1188 (7.4%) total AGSN DCC enrollees, and 412/2042 (20.2%) non-AGSN enrollees. A smaller fraction of the AGSN population threatens internal validity to the Niterói AGSN DCC population, but we were unable to visit six AGSN DCCs due to safety concerns.

Our study assessed point prevalence, so it was not possible to discriminate intermittent from persistent MRSA carriers. Persistent carriers are known to have higher bacterial loads, increased potential of transmission and are at higher risk of autoinfection [10, 30]. Furthermore, the rate of *S. aureus* colonization may be underestimated without simultaneous swabs of other body sites (e.g., axilla, groin, pharynx, and anus). Additionally neither employees of the DCCs, nor children’s family members were sampled. Despite these limitations, this study revealed the importance of DCCs, especially those located in informal settlements as a reservoir and potential nidus of transmission of MRSA.

## Conclusion

The disaggregation of health data by census tracts provide detailed neighborhood-specific health information. Targeted health policies can be designed to address disparities between communities with this type of information. Consistent with other studies of socially isolated groups, those in the most vulnerable communities had a higher prevalence of both *S. aureus* and MRSA colonization in comparison to children attending DCCs in non-informal areas. These data suggest that transmission of *S. aureus* and MRSA is occurring in DCCs rather than at home, although more accurate geocoding will be required to identify where transmission occurs. Further studies assessing the clonal diversity of *S. aureus* and MRSA in closed communities are necessary to thoroughly understand how isolation influences both carriage and transmission*.* Our study highlights the importance of DCCs located in slums as potential reservoirs for MRSA, and calls attention to the need for targeted surveillance in such communities.

## Authors’ information

EDVB: MPH. School of Medicine, Programa de Pós-graduação em Ciências Médicas, Departamento Materno Infantil, Fluminense Federal University, Niterói, Rio de Janeiro, Brazil.

FAA: PhD. Laboratório Universitário Rodolpho Albino, Programa de Pós-graduação em Patologia, Fluminense Federal University, Niterói, Rio de Janeiro, Brazil.

MFNF: BSC. Laboratório Universitário Rodolpho Albino, Fluminense Federal University, Niterói, Rio de Janeiro, Brazil.

MOS: School of Medicine, Departamento Materno Infantil, Fluminense Federal University, Niterói, Rio de Janeiro, Brazil.

TVC: School of Medicine, Departamento Materno Infantil, Fluminense Federal University, Niterói, Rio de Janeiro, Brazil.

RES: MPH. University of California, Berkeley, Division of Epidemiology, School of Public Health, Berkeley, California, USA.

VAA: MD. School of Medicine, Programa de Pós-graduação em Ciências Médicas, Departamento Materno Infantil, Fluminense Federal University, Niterói, Rio de Janeiro, Brazil.

MAM: PhD. University of California, Berkeley, Division of Epidemiology, School of Public Health, Berkeley, California, USA.

LWR: MD. University of California, Berkeley, Division of Infectious Diseases and Vaccinology and Division of Epidemiology, School of Public Health, Berkeley, California, USA.

SS: PhD. School of Medicine, Fluminense Federal University, Departamento de Doenças Infecciosas, Niterói, Rio de Janeiro, Brazil.

LES: MsCi. School of Medicine, Fluminense Federal University, Programa de Pós-graduação em Patologia, Niterói, Rio de Janeiro, Brazil.

CAAC: PhD. School of Medicine, Programa de Pós-graduação em Ciências Médicas, Departamento Materno Infantil, Niterói, Rio de Janeiro, Brazil.

## Authors’ original submitted files for images

Below are the links to the authors’ original submitted files for images. Authors’ original file for figure 1 Authors’ original file for figure 2

## Abbreviations

*S. aureus*: *Staphylococcus aureus*
MRSA: Methicillin-resistant *Staphylococcus aureus*
CA-MRSA: Community-associated methicillin-resistant *Staphylococcus aureus*
HC: Healthcare
DCC: Day Care Center(s)
AGSN: *Aglomerado subnormal*/Subnormal agglomerate.
