# Supplementary figures and images for: High prevalence of Staphylococcus aureus and methicillin-resistant S. aureuscolonization among healthy children attending public daycare centers in informal settlements in a large urban center in Brazil
Source: BMC Infect Dis. 2014 Oct 6;14:538. doi: 10.1186/1471-2334-14-538 (PMC4287590; doi:10.1186/1471-2334-14-538)

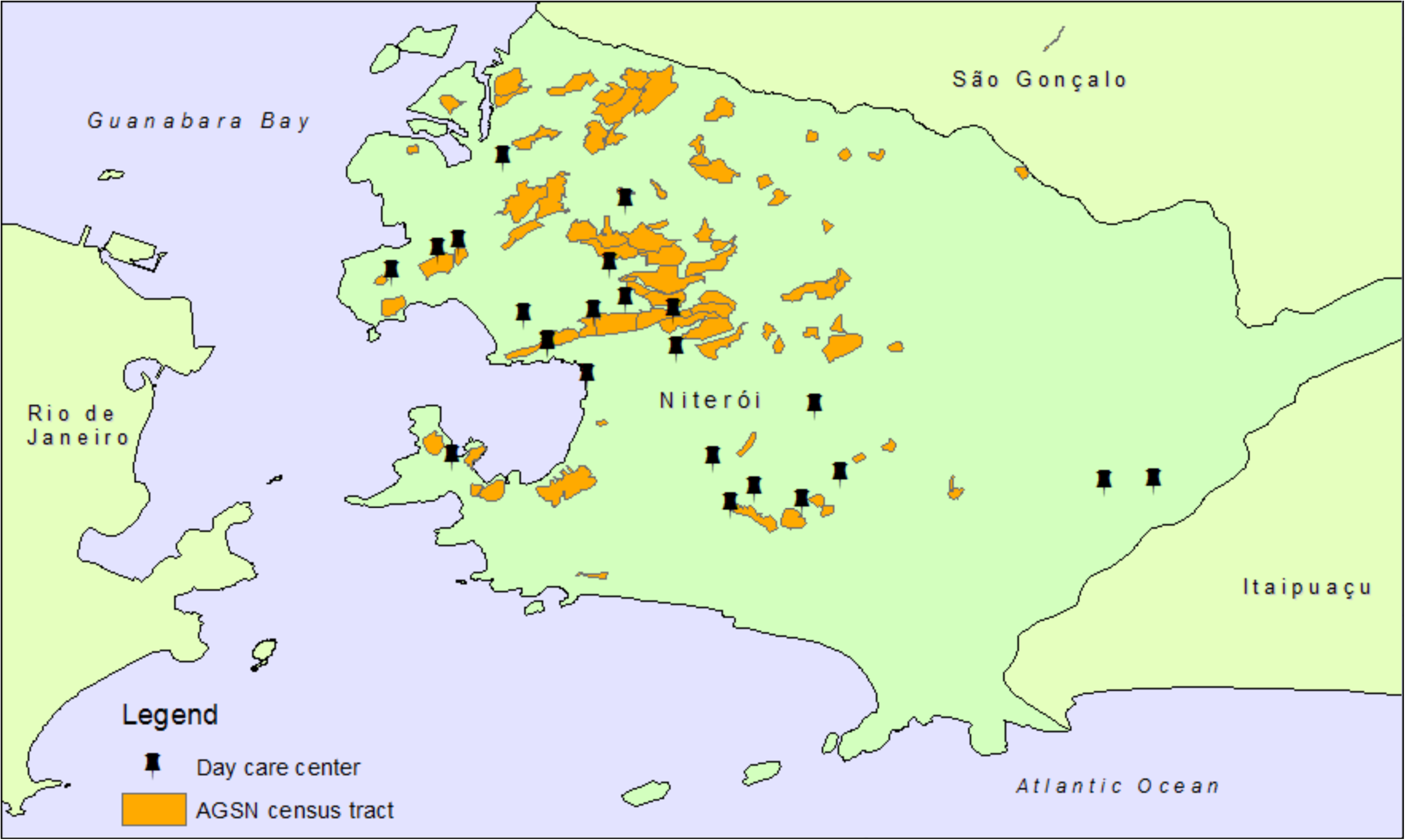

Supplement: Supplementary file 1 — Authors’ original file for figure 1 [file 12879_2014_3995_MOESM1_ESM.tiff]

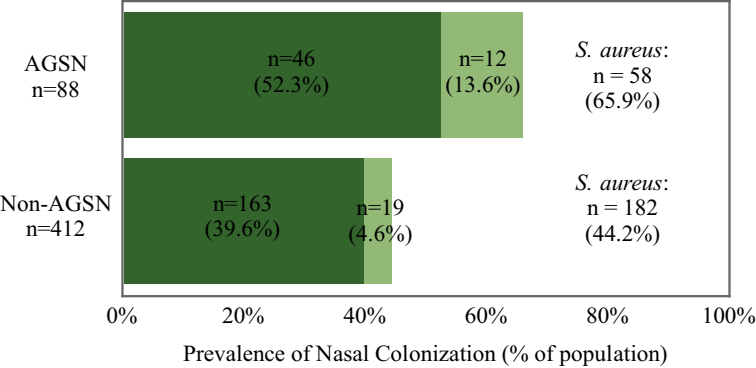

AGSN  
n=88

n=46  
(52.3%)

n=12  
(13.6%)

*S. aureus*:  
n = 58  
(65.9%)

Non-AGSN  
n=412

n=163  
(39.6%)

n=19  
(4.6%)

*S. aureus*:  
n = 182  
(44.2%)

0%

20%

40%

60%

80%

100%

Prevalence of Nasal Colonization (% of population)

Supplement: Supplementary file 2 — Authors’ original file for figure 2 [file 12879_2014_3995_MOESM2_ESM.pdf]
